# Reliably calibrating X-ray images required for preoperative planning of THA using a device-adapted magnification factor

**DOI:** 10.1371/journal.pone.0307259

**Published:** 2024-08-22

**Authors:** Heinrich Brüggemann, Aksel Paulsen, Ketil Oppedal, Markus Grasmair, Dietmar Hömberg

**Affiliations:** 1 Department of Orthopedic Surgery, Stavanger University Hospital, Stavanger, Norway; 2 Department of Public Health, The Faculty of Health Sciences, University of Stavanger, Stavanger, Norway; 3 Radiology Department, Stavanger University Hospital, Stavanger, Norway; 4 Department of Electrical engineering and computer science, University of Stavanger, Stavanger, Norway; 5 Department of Mathematical Sciences, Norwegian University of Science and Technology, Trondheim, Norway; 6 Weierstrass Institute, Berlin, Germany; University of Memphis, UNITED STATES OF AMERICA

## Abstract

**Background and aim:**

Calibrated pelvic X-ray images are needed in the preoperative planning of total hip arthroplasty (THA) to predict component sizes. Errors and mismatch in the size of one or more components are reported, which can lead to clinically relevant complications. Our aim is to investigate whether we can solve the fundamental problem of X-ray calibration and whether traditional X-ray still has a place in preoperative planning despite improved radiological alternatives.

**Methods:**

Based on geometric and radiographic principles, we estimate that the magnification factor is adapted to the X-ray device and depends strongly on the source-image distance of the device. We analyse the errors of the various calibration methods and investigate which narrow range can be expected to show that the center of rotation is sufficiently accurate. Based on the results of several CT-scans we defined an adapted magnification factor and validated the degree of measurement accuracy.

**Results:**

The true magnification of objects on X-ray images depends mainly on the device settings. Stem size prediction is possible to a limited extent, with an error margin of 4.3%. Components can be predicted with a safety margin of one size up and down as with CT or 3D images. The prerequisite is that the source-image distance is greater than or equal to 120 cm, the table-image distance is known, and the object-image distance is estimated according to the patient’s BMI. We defined a device-adapted magnification factor that simplifies the templating routine and can be used to obtain the most reliable preoperative dimensional measurements that can be expected from X-ray images. We found the error margin of the magnification factor with the highest degrees of prediction and precision.

**Conclusion:**

Preoperative planning is reliable and reproducible using X-ray images if calibration is performed with the device-adapted magnification factor suggested in this paper.

## 1. Introduction

Despite technological advances in radiological imaging, such as CT, MRI and 3D applications [1–3], digital X-ray images of the pelvic and the hips are still relevant in preoperative planning of prosthetic surgery. These images are a valuable resource for the surgeon to assess the extent of the damaged hip and to recognize intraoperative challenges [4, 5].

Surgeons use templating applications for preoperative planning to visualize how to restore the anatomy and kinematics of the damaged hip. They determine appropriate component sizes, offsets and angles [6]. This planning provides valuable insight into the type and size of components needed intraoperatively and ensures stock ordering and information flow for the entire surgical team.

However, the use of X-ray-based calculation is controversial in the literature due to their limited accuracy and reliability [7, 8]. X-ray images always show objects at an enlarged scale and therefore dimensional measurements of an object, in this case the hip, are not easily possible on X-ray images without calibration. Unlike in a CT scan, in a two-dimensional X-ray image, the real size of an object depends on its distance from the X-ray source to the image-plate, called source-image-distance (SID), and the object-image-distance (OID). The calibration is done with a magnification factor, which in turn can be calculated if all variables are known. However, this is not the case for the hip-to-table distance (OTD) which is a subset of OID and is unknown.

The OTD or more precise, the distance of the center of rotation (COR), that is, the middle of the hip joint (object), to the table is a patient- dependent variable that can only be determined indirectly. COR is not accessible for measurements and OTD can therefore only be estimated. This makes calibration a critical step that various calibration methods have tried to solve.

Object-based methods like the use of external calibration marker (ECM) with a known size, as a reference is the current standard for calibration [9, 10]. Alternatives are the use of a fixed magnification factor, on-screen graphical calibration, or even the traditional analog acetate overlay technique. All methods have a range of errors determining the true magnification factor [7, 8, 11, 12].

ECMs require exact placement relative to the femoral head and the COR, a shortcoming that makes this method susceptible to human and technical errors [7, 9, 13]. All other competing techniques like fixed magnification factor have the same challenge in identifying the coordinates of the COR which causes inaccuracies in the determination of the magnification factor [11, 14].

Inaccurately calibrated X-ray images have led to discrepancies between predicted and implanted components, called mismatch, which is believed to be the main cause of under- or overestimated component sizes. Calibration errors have resulted in clinically complications such as subsidence, intraoperative periprosthetic fractures, leg length discrepancy and instability [15–19]. Therefore, according to [8], templating results are no longer considered suitable for the precise prediction of implant components but should only be used as a guiding principle [8, 20].

Reliable measurement accuracy in X-ray images is required for preoperative planning, but not solved satisfactorily. The competing methods of CT and 3D applications can easily display a true-to-scale image of the pelvis and hip joints [2, 3, 21]. However, these alternative methods are not available to all departments and are not readily approved in all countries due to the increased radiation exposure.

Our aim is to investigate whether we can solve the fundamental problem of X-ray calibration and whether traditional X-ray still has a place in preoperative planning despite improved radiological alternatives. We want to determine what prerequisites must be applied so that the reliability and reproducibility of preoperative templating on calibrated X-ray images is sufficiently large, that this approach can still be used as an alternative to CT and 3D imaging.

## 2. Methods

### 2.1 Geometric and radiographic principles

**Fig 1:**
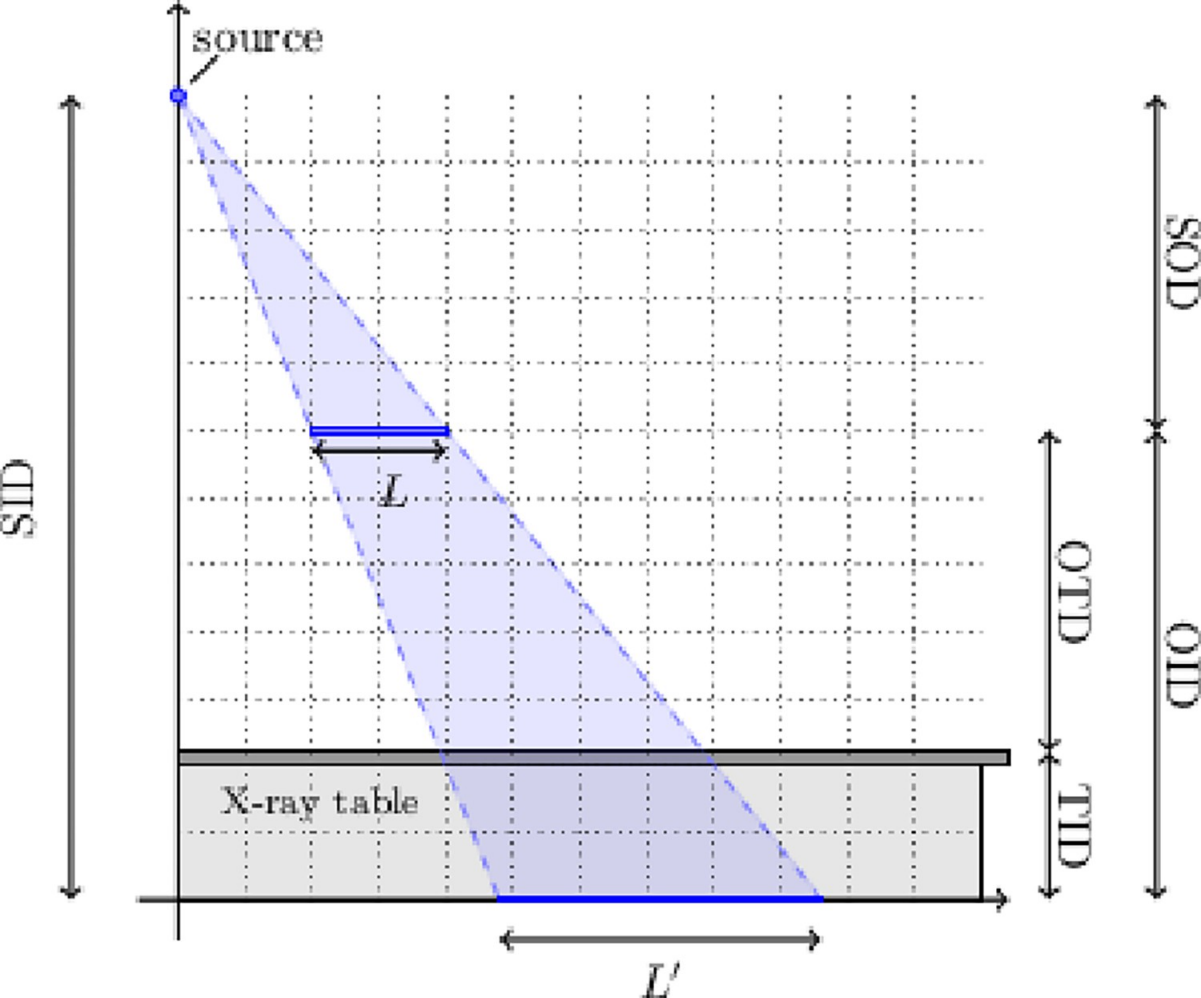
Graphical representation of the variables. The source, which is a point-shaped beam generator, sends a cone-shaped beam of radiation through the body (the object, L) onto the detector to obtain the image (L’). The source-image distance (SID) is the sum of the source-object distance (SOD) and the object-image distance (OID), which in turn is the sum of the object-table distance (OTD) and the table-image distance (TID).(M. Grasmair).

In the following, we will discuss some basic geometric and radiographic principles to explain how the variables are defined mathematically and graphically and which of them are important for calculating the magnification factor (Fig 1).

X-rays are created by a point-shaped beam generator (source) that sends a cone-shaped beam of radiation through the body (object) onto the detector to obtain the image. The patient lies flat on the table or stands in front of the table a few cm above/away from the image receptor. Here we will only consider the former situation of patients lying on the table. The object-image distance (OID) is the sum of the object-table distance (OTD) and table-image distance (TID), where OTD is patient-dependent and TID is device-dependent. Depending on the respective device, TID can vary from 2.5 cm up to 10 cm, including the thickness of the table and mattress.

The magnification depends on the source-image distance (SID), i.e., the total sum SOD+ OTD+TID. Normally, the local device setting for SID can vary between 100 cm and 130 cm depending on the anatomy of the patient.

**Fig 2:**
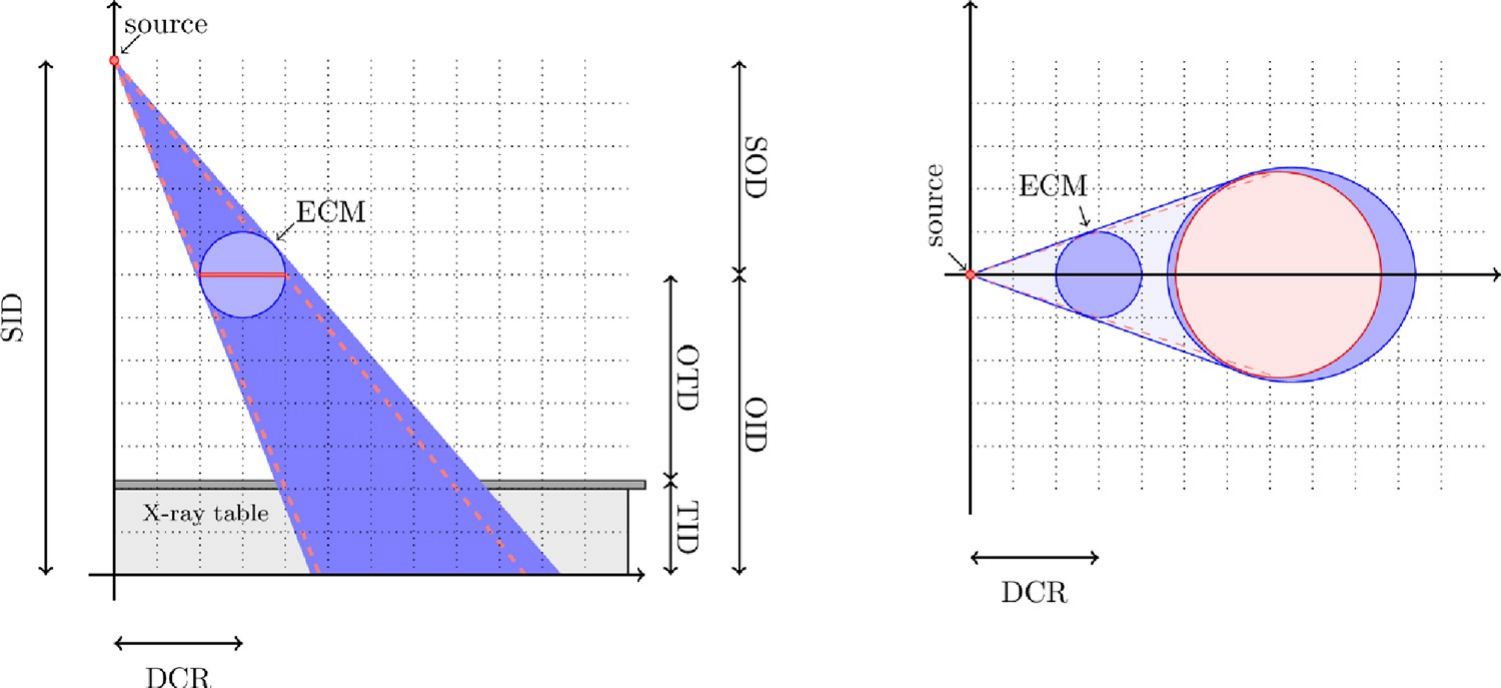
Geometrical principles. ((A) front view, (B) bird’s-eye view). The mapping of a spherical object (blue) is slightly larger in an image than a disc object (red) lying outside the central ray. The unit settings and the size and shape of the ECMs affect the magnification. If a spherical object (blue) is placed outside the central ray, it appears as an ellipse, where both the minor and major axes are larger than the diameter of a corresponding coin (red). (M. Garsmair).

The apparent size and shape of an object in an image depends on its placement relative to the central ray. If a spherical object or a disc is placed directly in the central ray, it appears as a circle in the image. If the object is placed outside the central ray (distance to the central ray (DCR) > 0), a spherical object appears as an ellipse, where both the minor and major axes of the spherical object are larger than the diameter of a corresponding disc (Fig 2) [16].

Most protocols use a standard SID setting between 100 cm and 120 cm. This is sufficient to map the lower part of the pelvis and the proximal part of the femur. If the pelvis is oversized, the SID setting has to be increased to 130 cm or more to fit the image on the image plate.

The intercept theorem credited to the ancient Greek mathematician Thales governs the magnification m. This theorem states that the proportion between the true length *L of an object* in a plane with distance *z (OID)* from the image and the projected length *L’* in the image plane is the same as the ratio between *h (SID)* and *h–z* (SID-OID) (Fig 1), as shown in Eq (1):

$$m=\frac{h}{h-z}=\frac{L'}{L}=\frac{SID}{SID-OID}$$ Eq (1)

**Fig 3:**
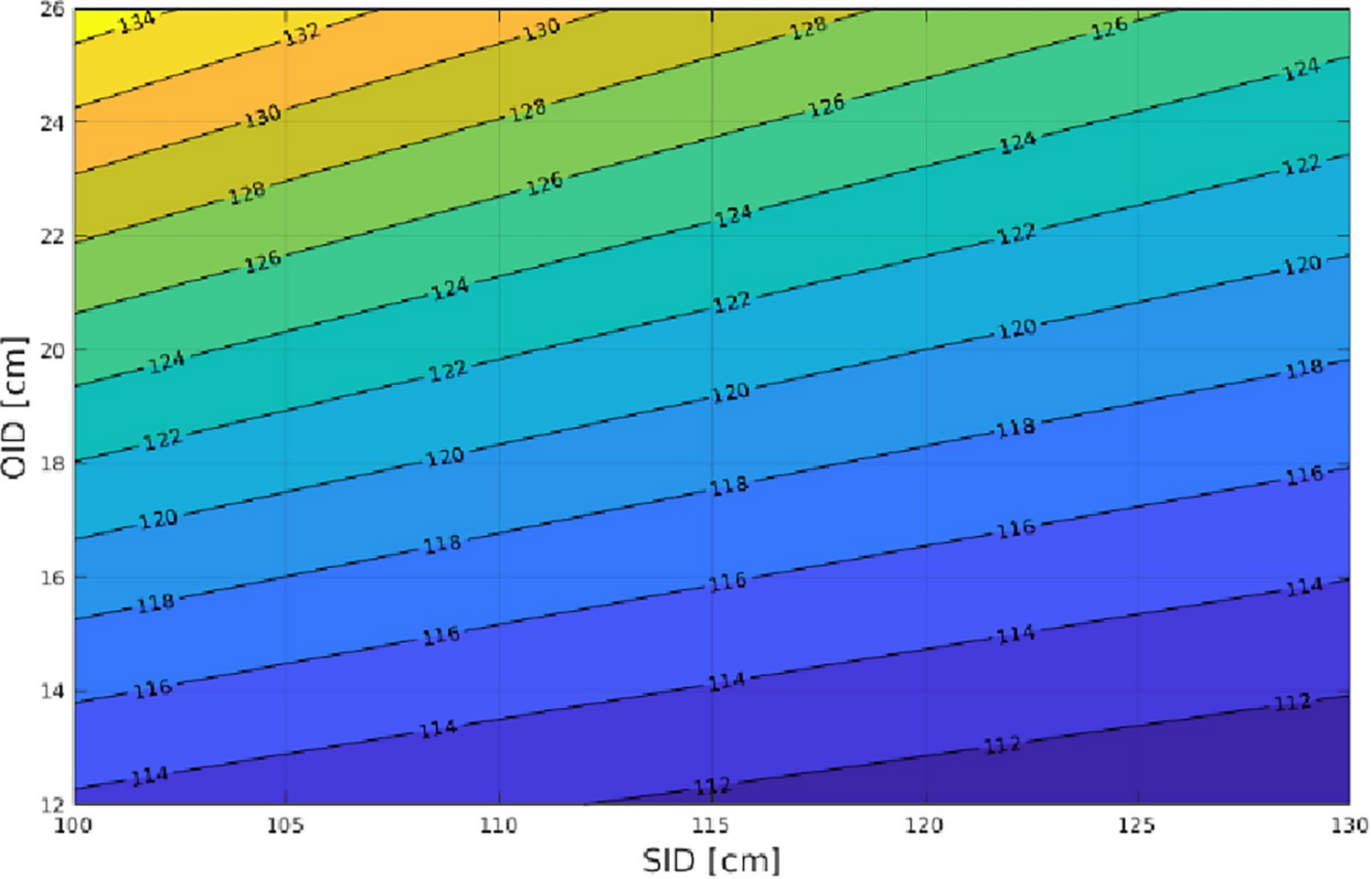
The percentaged magnification factor. The magnification factor m depends on h = SID and z = OID. For example, m = 120% when SID = 120 cm and OID = 20 cm.

Fig 3 shows the variation in magnification depending on SID and OID. For a fixed SID, the magnification increases as the object moves farther away from the image (OID). Practically, the size of a shadow increases as the object moves toward the light source. However, if the light source moves farther away from the image without the object moving, then the shadow of the object decreases. Alternatively, with a fixed OID, the magnification decreases as the source-image distance (SID) increases.

Here, the “object” is the hip, or more precisely, the COR. This means that for a fixed SID of 120 cm, the magnification increases as the COR moves farther away from the image (i.e., patient with hypertrophy of the glutes or extreme obesity). However, for a fixed COR, the magnification decreases as SID increases (i.e., the X-ray source is farther from the image).

### 2.2 Prerequisites

Objects in X-ray images are always enlarged (Fig 1) [22]. Theoretically, the real size and shape of an object can be calculated with the magnification factor if the coordinates of the depicted object in the peripheral beam path and the settings of the device are known precisely. This is not the case with the hip-to-table distance, since the coordinates of COR are unknown and thus OTD cannot be determined directly.

Our calculations are based on the following prerequisites: the digital radiograph image is either the size of a square plate (i.e., 40 cm x 40 cm) or a rectangle (i.e., 50 cm x 60 cm). The central beam is focused at a right angle over the symphysis of the symmetrically positioned lower pelvis of the patient. Both proximal femurs have an optimal internal rotation of 10–15 degrees. The SID is between 100 cm and 130 cm.

We use the information from the DICOM file format, in which all digitalized data are stored. The DICOM file format includes patient information and provides information on image characteristics including image resolution, which is determined by the pixel spacing. We assume that the typical digital radiograph image pixel spacing is 0.2 mm or lower.

We are interested in templating applications that identify ECMs either automatically using edge-detection techniques or manually. Calibration is performed according to the preset reference value by adjusting the measuring scale of the X-ray.

We refer to component templates of all kinds of stems that are imported into the templating applications and differ either symmetrically or laterally by 1–2 mm. The margin between two sizes is often not more than 1 mm for each size or 2 mm on one side for lateralization. Our calculations were only done for the femur component, since the results can be transferred directly to the acetabulum component.

## 3. Results

In this section we present the results of our error analysis of the different calibration methods and their influence on the templating results. The analysis is based on the data currently available in the literature. Our aim is to assure the calculation of an implant size with a tolerance of one size up and down and find the error range from which we can sufficiently determine the accuracy of the templating process.

### 3.1 Error analysis

By observing that a standard implant width is approximately 2 cm and that the difference in width between two consecutive implant sizes is approximately 1 mm, the relative error in the length measurements during templating must be below 5%. In the sequel, we will call calibration procedures leading to an error that stays below 5% safe calibration methods.

We have identified two main sources of error. On the one hand, measurement errors occur due to the limited resolution of the X-ray plate. However, there are also errors in the estimation of the magnification factor. Whereas the first error is independent of the choice of calibration method, the latter differs strongly between calibration methods with and without ECMs.

### Error due to resolution of X-ray plate

As discussed previously, measurements on the X-ray image can only be performed with a certain accuracy because of the limited resolution of the X-ray plate. For a long SID of 130 cm and a short OID of 14 cm, an object with a 20 mm diameter appears on the X-ray plate with a diameter of 22.4 mm. For a short SID (100 cm) and long OID (25 cm), the object appears with a diameter of 26.7 mm.

An object with a diameter of 20 mm will, with a pixel spacing of 0.2 mm, have a size between 112 and 134 pixels in the X-ray image. If we assume a measurement error of one pixel, we expect a relative error of at least 0.7% in diameter, which leads to an error of approximately the same size in all further length measurements. In order to reach an accuracy of 5%, the remaining errors must therefore be smaller than 4.3%.

### Errors for a fixed magnification factor

As shown above, the templating will be sufficiently accurate, if the total errors stay below 4.3%. We have performed an error analysis based on the theorem of Thales (Eq (1)). The detailed derivation of these results shows that there is a narrow range (safe zone) where OID can be expected, if the fixed magnification factor and the device-specific SID and TID are known (see S1 Section in S1 File and Table 1).

**Table 1:** Example for the safe zones of OID. A fixed magnification factor m = 120% and a standard SID of 120 cm and TID of 7.5 cm will yield sufficiently accurate results for OID in a safe zone between 15.5 cm and 24.1 cm. COR will occur within this zone with a calibration error of 4.3%. (H. Brüggemann).

* *		*SID*				* *
		*100 cm*	*110 cm*	*120 cm*	*130 cm*	
*Fixed magni-fication*	*115%*	*9*,*1–16*,*6 cm*	*10*,*0–18*,*3 cm*	*11*,*0–20*,*0 cm*	*11*,*9–21*,*6 cm*	*OID range*
	*120%*	*12*,*9–20*,*1 cm*	*14*,*2–22*,*1 cm*	*15*,*5–24*,*1 cm*	*16*,*8–26*,*1 cm*	
	*125%*	*16*,*4–23*,*3 cm*	*18*,*0–25*,*6 cm*	*19*,*7–28*,*0 cm*	*21*,*3–30*,*3 cm*	

### Errors with object-based calibration

To estimate the magnification factor, one measures the diameter of the reference object in the image, in this case, of the ECM. If a single ECM is used for calibration, two error sources can generally be identified, and one additional error source may occur in the case of spherical markers.

The first error source is measurement errors of the ECM on the X-ray plate. The resolution error mentioned above occurs again when we are measuring the size of the ECM. An ECM with a diameter of 30 mm will appear on the X-ray plate with a diameter between 33.6 mm and 40 mm. With a pixel spacing of 0.2 mm, it thus will have a size of between 168 and 200 pixels on the X-ray. If we assume again a measurement error of one pixel, we therefore have to expect a relative error of at least 0.5% in the diameter of the marker, which then leads to an error of approximately the same size in all further length measurements. Considering the errors discussed above, the remaining errors have to be smaller than 3.8% for safe calibration.

The second error source is the placement of the marker in the wrong plane. For a detailed derivation of these results, refer to S2 Section in S1 File. For a typical OID of approximately 20 cm and an SID of 120 cm, an ECM has to be placed with a vertical distance of at most 3.8 cm from the COR to meet the requirements for safe calibration; for an SID of 100 cm, this is reduced to a maximal vertical positioning error of 3.0 cm.

The horizontal placement of the ECM can have an additional influence on the error (S3 Section in S1 File). Specifically, for a spherical ECM and an SID of 120 cm and an OID of 20 cm, a potential additional error of 0.5% is obtained when DCR = 10 cm which even increases to 2.0% when DCR = 20 cm. We stress again that the specific effect of this type of distortion depends on the templating software and the above examples represent only the worst case errors.

The error analysis in the case of coin-shaped markers is more complicated, and the results depend on the placement of the coin. If the coin is placed horizontally, these errors do not occur; otherwise, the error may increase up to that obtained for a spherical marker.

**Fig 4:**
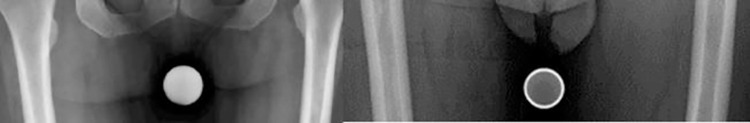
Different mapping effects of a spherical ECM. The mapping of a spherical ECM of metal can vary from a normal exposed circle (left) to a circle with a “halo ring phenomenon” (right) caused by the reflection of different materials and different radiation. (H. Brüggemann).

**There are several other factors** that can affect the magnification factor. Examples are an incorrect X-ray beam angle, wrong focus or an asymmetric position of the pelvis on the X-ray table. Extra equipment, such as a thick comfortable mattress on the X-ray table, affects the OID if it is not included in the device-specific OID. The mapping of a spherical ECM of metal can vary from a normal exposed circle up to a circle with a “halo ring phenomenon” (Fig 4).

It is unknown how templating software manages different mappings with automatic detection.

Finally, templating programs perform calibration without any dimensional changes in the X-ray image itself. There are no corrections for geometrical distortion and error, only the scale is changed. The objects in the X-ray image retain their original appearance.

### 3.2 Safe calibration with a device adapted fixed magnification factor

To achieve sufficient accuracy for OTD in a safe zone, we use external data from several studies to find the most realistic OTD based on the range of COR for most of the patients including those who are not in the common range of BMI (according to WHO 18.5–29.9 kg/cm2). Based on a study of 50 pelvic CT scans in [23], OTD ranged from 7.9 cm– 14.2 cm. Similarly, in a study of 398 pelvic CT scans in [24], OTD ranged from 8.9 cm– 16.6 cm. A larger range of values from 6.7 cm– 16.8 cm was given in [25] based on 100 pelvic anteroposterior X-rays with an internal calibration marker (ICM). Additionally, Clarke et al. [22] found a range for the OID from 12.5 cm– 20.5 cm based on 414 pelvic X-rays. However, these results were obtained with X-rays taken in a standing position and thus are expected to display larger variation (Table 2).

**Table 2:** OTD range from external data. (see in References) (Heinrich Brüggemann).

	Radiographs	Position	Number of patient	OTD range	range length	Mean OTD	Mean OID
Clarke IC et al. [22]	X-ray	Standing	414	8.0 cm– 16.0 cm	8,0 cm	12	19,5
Kulkarni A et al. [23]	CT	supine	50	7.9 cm– 14.2 cm	6,3 cm	11,7	19,2
Boese CK et al. [24]	CT	supine	398	8.9 cm– 16.6 cm	7,7 cm	12,75	20,25
Boese CK et al. [25]	CT	supine	100	6.7 cm– 16.8 cm	10,7 cm	11,75	19,25

In the following, we will only use the measurements from [23, 24]. Both of these are CT data with robust reference values for OTD; in the case of [24], the values of the OTD have been computed from the magnification factors provided therein. In the axial slices, a horizontal line through both COR is drawn and the vertical line from the symphysis through os sacrum where OTD is measured from the cutting point to the tabletop. All CT scans were taken in the supine position and thus are closer to the setting that we are most interested in.

Combining the ranges given there, we can expect for most of the patients an OTD between 7.9 cm and 16.6 cm. In our case, with a known TID of 7.5 cm, we obtain an OID range from 15.4 cm—24.1 cm. Comparing this with the margins given in Table 1, we see this almost perfectly coincides with the margins for a magnification factor of 120% and an SID of 120cm (15.5 cm– 24.1). Safe calibration can generally be ensured with these settings.

As we see from Table 1, the range of OIDs for which a fixed magnification factor yields a sufficiently accurate result depends strongly on the SID. Thus, it makes sense to choose a magnification factor that is adapted to the geometric configuration of the X-ray device. According to Eq (1) and observing that the OID is the sum of the OTD and TID, the true magnification factor can be computed, as shown in

$$m=\frac{SID}{SID-OTD-TID}$$ Eq (2)

When X-rays are taken, both SID and TID are known; the only unknown variable in this equation is OTD. We therefore propose using Eq (2) with an estimate of the OTD (or COR) to define an adapted magnification factor.

Based on the data in [23, 26] a narrow range of typical values of OTD can be determined. In the study [23], a mean value of 11.7 cm with a standard deviation of 1.3 cm was observed for OTD. Moreover, more than 85% of the measured OTDs were within the range of 10 cm-14 cm. From the study [26], the OTD statistics can only be inferred approximately. Thus, we will use the results of [23], and we propose the magnification factor

$$m=\frac{SID}{SID-\hat{z}}\;\hat{z}=TID+11.7\mathrm{cm}$$ Eq (3)

With an argumentation similar to the derivation of Eq (11) in S2 Section in S1 File, the error resulting from this magnification factor will remain within a desired tolerance *tol* if the condition

$$|OID-\hat{z}|\leq (SID-OID)tol$$ Eq (4)

is met. With a maximal tolerance of 4.3%, as discussed in Section 4.1, an SID of 120 cm and a TID of 7.5 cm, OTDs between 7.9 cm and 16.6 cm can be used. This encompasses all of the values measured in [23]. The largest values measured by [26], however, fall well outside this range. Additionally, for OTDs between 10 cm-14 cm, which include 85% of the values measured in [23], the maximal calibration error with this magnification factor is only 2.3%.

For an SID of 100 cm, the acceptable values of OTD are more restricted. Here, Eq (4) holds for OTDs between 8.1 cm and 15.0 cm. Thus, the extremal values in the studies [23, 26] fall outside this range. [27] used an SID of 40 inches (101.6 cm) and found a mean distance from the femur to the plate of OID = 15.3 cm (5.7 cm to 23.7 cm), corresponding to a previously reported mean height of 16.5 cm [27]. They only provided information on the OID, not which OTD was used. Therefore, these results are not comparable to ours. However, the range of 10 cm-14 cm still leads to the desired precision, with a maximal calibration error of 2.9%.

### 3.3 Findings and insights

Our investigation shows that an adapted fixed magnification factor that is based on a standardized SID of 120 cm or longer and a known TID leads to an error margin of only 4.3% and thus to a high degree of predictability and reliability relative to the choice of implant dimensions. The measurement results are not affected by additional errors that occur when using ECMs, different device settings or uncontrollable technical and human errors. This makes the calibration reproducible in the safe zone of known error limits. Based on this calibration approach, a sufficient prediction accuracy of one size up and down is achieved, making this method acceptable for the preoperative planning of THAs in everyday clinical practice.

TID and SID are important for the computation of a fixed magnification factor of the local device. We implement it in the magnification formula (Eq 2), which considers this device-dependent connection. To use our formula, TID must be collected from the corresponding device, and SID needs to be standardized. In our example with a SID = 120 cm and TID = 7.5 cm (including the mattress) and a fixed magnification factor of 120%, OID (COR) will be located in the safe zone between 15.5 cm and 24.1 cm (Table 1) above the X-ray plate and we can expect a sufficiently correct calibration. The safe zones for OIDs overlap widely at the different SIDs and fixed magnification factors (Table 1). This ensures a lower susceptibility in the accuracy of the calibration even with larger deviations from OTD with extreme BMI.

But OTD can be measured individually with a measuring stick in patients who are not in the BMI range of 18.5 to 35 (WHO) and the individual magnification factor can be calculated according to Eq (1) (z = OTD + TID) if wanted. Alternatively, SID can be increased or decreased by 10cm to correct biometric deviations such as height, weight or BMI. Even with extreme OTDs, a sufficient calibration can be secured. For example, increasing of SID with high BMI is necessary to fit the pelvic on the image-plate. This change of SID will not cause calibration errors as the OID is still within the safe zone provided the device adapted fixed magnification factor stays the same (Table 1).

Table 3 shows the length of safe zones in cm. Here we find the shortest safe zone of 6.9 cm with the short SID of 100 cm and a fixed magnification factor of 125% whereas in contrast to that the longest safe zone of 9.7 cm is achieved with a fixed magnification factor of 115% and long SID of 130 cm. In practical terms, this means that with SID = 120 cm and a magnification factor of 120% and a known TID, most OTDs are in the safe zone.

**Table 3:** The range of the safe zone for OID. The shorter SID and the larger the fixed magnification factor, the smaller the range for the value of OID. On the other hand, a longer SID of at least 120 cm and a fixed magnification factor of (less than or equal to) 120% is convenient in relation to a larger safe zone. (H- Brüggemann).

* *		***SID***				* *
		***100 cm***	***110 cm***	***120 cm***	***130 cm***	
***Fixed magni-fication***	***115%***	*7*.*5 cm*	*8*.*3 cm*	*9*.*0 cm*	*9*.*7 cm*	***Range of OID***
	***120%***	*7*.*2 cm*	*7*.*9 cm*	*8*.*6 cm*	*9*.*3 cm*	
	***125%***	*6*.*9 cm*	*7*.*6 cm*	*8*.*3 cm*	*9*.*0 cm*	

This device adapted calibration method simplifies the X-ray taking protocol and is more comfortable for the patients. Our formula is reliable and reproducible for templating X-ray images as calibration can be performed on all types of devices and we know the range of error. As far as we know, it is currently the safest calibration method that covers most hips regardless of sex and BMI. We have solved the limited possibilities of X-ray images and the uncertainty of determining the coordinates of COR in such a way that we know what errors we can allow ourselves and are therefore able to make the best of it. With our formula, preoperative templating on X-ray images allows a high degree of predictability of the size of the femoral component within one size up and down provided a caliber jump of 1–2 mm of the imported stem templates. Moreover, with the same formula, the acetabulum component can be determined safely.

### 3.4 Implementation

X-rays, despite alternatives such as CT and 3D imaging, can still be the gold standard in preoperative planning if the following insights are considered and implemented.

Here we present the framework for the successful implementation and daily use of our device adapted fixed magnification factor, which excludes masked errors, and which is based on the above accuracy requirements.

1. Avoid using an external calibration marker.
2. Measure TID, if necessary, include the tabletop and mattress thickness.
3. Define a standardized SID, adjust it to at least 120 cm or longer and note the value.
4. Choose a large safety zone for each X-ray device, depending on the TID, SID and fixed magnification factor.
5. If the patient is within the BMI range of 18.5 to 40, compute the fixed magnification factor using
$$\mathrm{Eq}.(3),i.e.,\;m=\frac{SID}{SID-\hat{z}}\;\hat{z}=TID+11.7\mathrm{cm}$$
  where SID and TID are the adjusted values (in cm).
6. If the patient is not within the BMI range of 18.5 to 40, estimate the OTD using a ruler and compute the magnification factor by
$$\mathrm{Eq}.(2),i.e.,\;m=\frac{SID}{SID-OTD-TID}$$
  In this case, radiologists should mark the estimate OTD and changes of SID on the X-ray image.
7. Depending on the templating application, insert the magnification factor directly or use a graphic method (draw a line with the length of the magnification factor (120% = 12.0 cm) and calibrate it to 100%).

## 4. Discussion

### 4.1 Critical review

The challenge of reliably calibrating X-ray images and precisely predicting component sizes are not solved [8, 26, 28]. However, there is no consensus on the accuracy of the measurement results from the preoperative planning [28]. The real coordinates of the OTD are neither known nor evident on two-dimensional X-ray images. Therefore, there remains a risk of incorrect calculation of the true magnification of X-rays images and their clinical consequences [11].

As shown above, there are three main groups in the literature investigating possible solutions. One group uses object-based calibration [16, 29–31]. The second one uses direct calculation with a fixed magnification factor [11, 15, 24, 32, 33] and the third group uses CT and 3D technology [1, 2, 21].

The first group, in which X-ray images are calibrated with an object of known size, is the most established and is considered to be the current gold standard [26], but a number of papers have shown that this calibration is more vulnerable than others, mainly because of error-prone technical and human errors like the uncontrollable placement [8, 15, 28]. It is not clear why it is the gold standard, although this method is clearly described in the literature as less accurate for calculating of the real magnification [8, 11, 26]. The results from this group are a relative comparison between planned and used component. The methods are not standardized. It is therefore not possible to draw any conclusions about the accuracy of the magnification factor.[6, 10].

Boese et al. conclude that the calibration error of external markers results in size differences of up to six sizes for the acetabular and four sizes for the femoral component [26]. Other examples for this disagreement are the mean error of 5.8% obtained by Baxter et al. [29], 6% by Archibeck et al. [7] and 6.8% by Sinclair et al. [8]. The same applies to the studies that retrospectively prove the calibration accuracy on internal objects of postoperative X-ray images [8, 9, 31, 34, 35]. However, our error analysis also shows that calibration with ECM is error prone and does not make any direct statement about the real magnification factor. This is different to our device adapted factor which is directly related to the real magnification.

In the second group, the calibration method with a fixed magnification factor is mainly based on SID [14]. Franken et al. compared object-based calibration methods with a fixed magnification factor of 121% and a locally defined SID. They found that the fixed magnification factor was the most reliable and efficient calibration method at that time [11]. Brew et al. used a mean magnification factor of 119.8% (range 117% - 123%), which was determined for the local radiology department (SID of 130 cm) [32]. Archibeck et al. did not detect a statistically significant difference in accuracy by using one method over the other [15]. Leo et al. (2015) showed that the absolute and relative errors using a fixed magnification factor were significantly smaller than those with ECM [33].

Olmedo et al. found it difficult to compare ECM studies with fixed magnification factor results because of non-standardized SID and methodology [28]. Hornova et al. previously recognized that the unit settings, precisely the variation of the SID and TID, are of central importance and influence of the magnification [17].

Referring to our methods, TID plays an essential part in the geometrical principles of magnification and is a device-dependent variable. The effect of TID on the magnification was taken into account only in some studies [12, 17, 28]. This effect was already described, but not implemented in a standardized templating protocol. In studies using the same SID, different magnification factors can occur because of different TIDs in the same department. It is important to know and to include the exact distance of the X-ray plate (image) under the table (including the mattress) to obtain the correct TID. For example, if SID is fixed to 100 cm, but TID is only 2.5 cm, the mean OID is 14.2 cm. If the TID is 10 cm, the mean OID is 21.7 cm, which will increase the magnification for the same SID.

In the third group, three-dimensional templating is based on CT scans and 3D technology. These images are calibrated automatically, and subsequent measurements are accurate. The role of CT and 3D printing in preoperative planning and surgical decision making has its clear advantages in better visualization and planning of the position of components, which is crucial in cases of complex THA. Precise planning of the component sizes and virtual placement is possible at once because of precise calibration and better pelvic orientation [15, 23, 26–30, 36]. However, better predictability is not guaranteed. In the review by [2] and the studies by [1, 3, 21, 36], the conclusion is that there still remains a difference of 2 mm between the templated and the implanted components. This is the same safe zone that we found for the calibration of X-ray images by using our adapted magnification factor [2]. We conclude that the predictability of component sizes from these resource-intensive applications is comparable to our method. In both cases, components can be safely predicted one size up and down. In the cost-benefit balance, this must certainly be taken into account.

Our approach differs from group one and two. With a device specific fixed magnification factor of for example 120%, a standardized SID of 120 cm and known TID we can ensure that the COR of patient is in the vertical safe zone between 15.5 cm and 24.1 cm (Table 1). This gives surgeons objective control over the quality of the calibration within a defined error range.

In all three groups mentioned above, most papers try to prove the accuracy of the magnification with the agreement of the implanted components and the measurement results [8, 12, 28, 35, 37]. This methodology ignores the effect of intraoperative decisions regarding individual challenges that need adjustment of the component size [26]. However, it is generally accepted to prove the accuracy of a calibration method with the implanted component sizes. Although this is obviously a surrogate for a direct assessment of the accuracy of a calibration method, it is the usual evidence presented [26].

A highly precise prediction of components does not simultaneously mean that it is also the best individually adapted component for restoring the kinematics intraoperatively. The final selected size of the components depends on the individual intraoperative circumstances, such as the bone stock, extent of joint destruction and implant positioning and intraoperative stability. However, we join with [8, 28], who rightly point out that the comparison between predicted and the finally implanted components can never be an argument for the accuracy of a calibration method but gives only somewhat realistic expectations.

An important question is how relevant the predictability of component sizes is from a clinical perspective in general. In fact, the size of a cemented prosthesis component must be less precise than that of the uncemented one. In practical terms, this means that the measurement accuracy is more important for the uncemented components requiring a press fit effect.

A worst-case scenario is that reaming the femur canal stops already several component sizes below the predicted size. It is unclear whether the reason is a measurement error from templating, a technical or a patient-dependent problem. If the surgeon sticks to the plan, this can lead to intraoperative periprosthetic proximal femur fractures, which are caused by an over-dimensioned predicted stem [19]. This is one possible explanation for intraoperative periprosthetic proximal femur fractures with uncemented femoral components [27, 38, 39]. In addition, there are few studies about the impact of calibration errors on long-term results and clinical outcomes [40].

In [41], the authors found a convenient method to determine the magnification factor by placing a coin on the radiograph plate (image). A coin that is adapted for a specific device and depictured a known size on the x-ray as if it were at the level of the center of rotation. This method seems to be less prone to errors and is more comfortable for patients and radiographers. The use of a variable distance for SOD of 90–120 cm and unknown TID allow this method to be device independent.

The influence of BMI and body shape is discussed controversially and there is no consensus so far. One of several reasons is the problem mentioned earlier, the variation in the methodology of the investigations. Some authors prefer to determine SOD, others the distance between the beam source and the symphysis or spina iliaca anterior superior. Again, others refer to OTD without considering TID. One group found no significant correlation between height, weight, BMI and vertical position of COR [28, 41–43], whereas the other group describe a weak to significant influence on the vertical shift of the COR [10, 11, 30, 31]. [41] illustrate by using CT scans that there is not much variation in the distance between the COR and image (OID) for BMI divergent patient. Our results show the same and with extreme BMI where failure is expected, manually determination of OTD can be used.

### 4.2 Strengths and weaknesses

We have rethought a fundamental problem and, with the help of Thales’ theorem, precisely described the error range that allows us to calculate predictability of the stem size. Using mathematics, we have objectified the calibration problems of X-ray images with an unknown variable based on a large population. Our formula is adjustable to any device and thus universally applicable. We have described and assessed the importance of the variables SID and TID and their impact on the magnification factor calculation.

As far as we know, no study has been able to show the strong influence of the device-dependent variation of the SID. Our protocol is less susceptible to human and technical errors. We have proven that the calibration error can be kept sufficiently small in a known safe zone and component sizes can be predicted with the greatest possible certainty regarding the limited resolution of X-ray devices.

A limitation is that we could not find any information about errors that occur during image creation and data processing that may affect the accuracy. For example, the attenuation or the intensity of the X-ray beams can both be reduced by absorption of tissue (depending on the thickness and homogeneity of the body tissue) or amplified by deflection and hyperreflexia causing artifacts. The sharpness of body structures and the quality of images depend on the intensity of the X-ray beam and their interaction in the body tissue [44]. Refraction, as known from light refracted in water, is normally neglected in X-rays due to the high photon energy, which gives the refractive index very close to one.

It is still unclear whether the X-ray beam, when passing through the body, behaves differently than outside the body due to interactions that can occur because of different tissue impedance. This aspect is not considered in previously published articles in which the size of an internal marker (ICM), such as a prosthetic head, is compared with that of the external marker (ECM) to prove the accuracy of the calibration [26].

Different templates of prosthesis components are integrated into templating applications and used either automatically from the software or manually by the surgeon using the proven overlay technique. There is little known about the quality and exact dimension and synchronization of those templates regarding the 1–2 mm differences.

Another limitation is that we did not assess the quality of the software. The templating application does not assess the quality of the X-ray images. The preoperative templating result is highly dependent on standardized X-ray images, in which the central X-ray beam is focused on the pubic symphysis, the pelvis is positioned symmetrically, the legs are internally rotated at 15 degrees and the required landmarks are clearly displayed.

Finally, our results apply only to the pelvic images taken in a supine position, not in a standing position. However, theoretically it is expected that the mean error in the standing position is at least greater than 4.3%.

## 5. Conclusions

The use of X-rays images for preoperative templating is still safe and acceptable. While calibration with ECM is the standard, it provides less accurate measurement results than calibrating with our device-specific fixed magnification factor.

We conclude that this calibration method ensures the same reliability and reproducibility with component prediction compared to CT scans and 3D reconstructions. It simplifies the X-ray routine for the radiographs and makes it more comfortable for the patient. Further experimental studies are required to verify the safe zone for all components.

## Supporting information

S1 File (DOCX)

## Abbreviations

BMI: Body mass index, normal value 18.5–35
COR: Centre of rotation
DCR: Distance from the central ray
DICOM: Digital imaging and communications in medicine
ECM: External calibration marker
Eq: Equation
err: Erro
h, SID: source-image distance
m: Magnification factor
OID: Object-image distance
OTD: Object-table distance
PACS: Picture archiving and communication system
r: Radiu
SID: Source-image distance
SOD: Source-object distance
THA: Total hip arthroplasty
TID: Table-image distance
Tol: olerance
z, OID: object-image distance
