# Supplementary material for: Reliably calibrating X-ray images required for preoperative planning of THA using a device-adapted magnification factor
Source: PLoS One. 2024 Aug 22;19(8):e0307259. doi: 10.1371/journal.pone.0307259 (PMC11340982; doi:10.1371/journal.pone.0307259)
Supplement: S1 File — (DOCX) [file pone.0307259.s001.docx]

# Supporting information

## S1 Error for fixed magnification factor

We consider the situation where a fixed magnification factor *m* is used for the calibration. We assume that we are imaging an object of length *L* with OID equal to *z* and SID equal to *h*. In this case, the image of this object has a length *L'* that is calculated by using

Eq. (5) as

on the X-ray plate. With the magnification factor *m*, this is interpreted by the templating tool to be a length *L''*,

Eq. (6)

The relative error is therefore equal to

Eq. (7)

Our accuracy requirement states that this relative error needs to be smaller than the given tolerance of tol=4.3%. For a fixed SID of *h*, this gives us the upper and lower bounds for the OID. If the OID is between

Eq. (8)

and

Eq. (9)

the relative length error will be within the required tolerance.

## S2 Safe positioning margins with an ECM

The usage of an ECM for calibration will lead to the magnification factor

Eq. (10)

where *z*_ECM_ is the distance between the ECM and the image. Inserting this value into Equation (5) and replacing *err* by *tol*, we find that the error remains within the tolerance tol if the following condition is met:

Eq. (11)

## S3 Distortion errors for ECMs

As noted in [16, 39], a spherical marker can appear on an X-ray as an ellipse (Fig 2). In such a case, the size detected by the templating software depends on the specific algorithm that is implemented. In the worst case, the apparent major axis *D_app_* of the ellipse is used as input for the scaling calculations. According to Equation (Eq 4) in [16], this can be computed as

Eq. (12)

This results in the error

Eq. (13)

With a marker radius r = 1.5 cm and a typical value of *h-z* greater than 70 cm, the first term in the approximation results in errors far below 0.1% and can therefore be ignored. In contrast, the second term can be significantly larger if the marker is placed far away from the central ray (at the edge of the X-ray plate). This may occur when placing the ECM lateral to the damaged hip or on the skin over the major trochanter or too far away from the urogenital area.
